# Discrimination of Low-Energy Acetabular Fractures from Controls Using Computed Tomography-Based Bone Characteristics

**DOI:** 10.1007/s10439-020-02563-4

**Published:** 2020-07-09

**Authors:** Robel K. Gebre, Jukka Hirvasniemi, Iikka Lantto, Simo Saarakkala, Juhana Leppilahti, Timo Jämsä

**Affiliations:** 1 Research Unit of Medical Imaging, Physics and Technology, University of Oulu, Oulu, Finland; 2 Department of Radiology & Nuclear Medicine, Erasmus University Medical Center, Rotterdam, The Netherlands; 3 Division of Orthopaedic and Trauma Surgery, Oulu University Hospital, Oulu, Finland; 4 Medical Research Center, University of Oulu and Oulu University Hospital, Oulu, Finland; 5 Diagnostic Radiology, Oulu University Hospital, Oulu, Finland

**Keywords:** Computed tomography, Machine learning, Acetabular fracture, Trabecular structure, Gray-level co-occurrence matrix

## Abstract

The incidence of low-energy acetabular fractures has increased. However, the structural factors for these fractures remain unclear. The objective of this study was to extract trabecular bone architecture and proximal femur geometry (PFG) measures from clinical computed tomography (CT) images to (1) identify possible structural risk factors of acetabular fractures, and (2) to discriminate fracture cases from controls using machine learning methods. CT images of 107 acetabular fracture subjects (25 females, 82 males) and 107 age-gender matched controls were examined. Three volumes of interest, one at the acetabulum and two at the femoral head, were extracted to calculate bone volume fraction (BV/TV), gray-level co-occurrence matrix and histogram of the gray values (GV). The PFG was defined by neck shaft angle and femoral neck axis length. Relationships between the variables were assessed by statistical mean comparisons and correlation analyses. Bayesian logistic regression and Elastic net machine learning models were implemented for classification. We found lower BV/TV at the femoral head (0.51 vs. 0.55, *p* = 0.012) and lower mean GV at both the acetabulum (98.81 vs. 115.33, *p* < 0.001) and femoral head (150.63 vs. 163.47, *p* = 0.005) of fracture subjects when compared to their matched controls. The trabeculae within the femoral heads of the acetabular fracture sides differed in structure, density and texture from the corresponding control sides of the fracture subjects. Moreover, the PFG and trabecular architectural variables, alone and in combination, were able to discriminate fracture cases from controls (area under the receiver operating characteristics curve 0.70 to 0.79). In conclusion, lower density in the acetabulum and femoral head with abnormal trabecular structure and texture at the femoral head, appear to be risk factors for low-energy acetabular fractures.

**Electronic supplementary material:**

The online version of this article (10.1007/s10439-020-02563-4) contains supplementary material, which is available to authorized users.

## Introduction

Low-energy acetabular fractures in the elderly often occur due to low impact traumas such as lateral falls from a standing height.^9^,^10^ Typical patterns of these fractures are displacement of the anterior column, anterior wall and anterior with posterior hemi-transverse fractures as well as anteromedial dislocation of the femoral head.^9^,^10^,^30^,^33^ According to a recent study, fall-related mortality for adults over 75 years of age in the United States of America (USA) increased dramatically from 51.6 to 122.2 per 100,000 people between 2000 and 2016, respectively.^19^ Low-energy acetabular fractures in the elderly also pose major health and socioeconomic concerns with possible treatment complications due to health conditions, osteopenia and associated femur head fractures.^16^ Mobility and housing dependence of persons with pelvic fractures has been shown to increase with a long-term decline in the physical quality of life.^4^ Identifying potential risk factors for low-energy acetabular fractures in the elderly may therefore be crucial to developing better diagnostic and treatment options.

Osteoporosis (OP) is associated with an increased risk of hip fractures^25^ and the clinical standard used to quantify OP is the measurement of bone mineral density (BMD) by dual energy X-ray absorptiometry (DEXA).^5^ For hip fractures, selective use of BMD in conjunction with other clinical risk factors has been proposed.^25^ Other prior studies have shown that the prediction of hip fractures is improved when other measurements such as hip geometry and trabecular bone architecture are included.^25^,^43^ However, for low-energy pelvic fractures, it is not known whether OP, pelvic and hip geometry, and/or trabecular bone architecture are risk factors.

A common imaging modality that is used to assess acetabular and other pelvic fractures is computed tomography (CT).^5^ However, the limited resolution of clinical CTs can be a disadvantage when performing image analysis and feature extraction.^6^,^39^ Previous CT based studies using relatively larger slice thickness (≤ 5 mm)^6^,^7^,^29^ and pixel spacing (≤ 1 mm)^38^,^44^ have demonstrated that it is possible to extract useful information under low-resolution settings. Hence, trabecular architectural features defined by trabecular structure, texture and density can be extracted from clinical CT.^1^,^26^,^39^ Bone quantity can be characterized by bone volume fraction (BV/TV).^39^ Trabecular texture can be analyzed using various methods such as fractal dimensions^24^ and gray level co-occurrence matrix (GLCM).^26^,^39^ In addition, bone mineral density can be also estimated from the first-order statistics of the gray value (GV) histogram within a 3D volume of interest (VOI).^22^,^26^ Moreover, bone microstructure measured from clinical CT has been shown to be associated with BV/TV,^1^ histogram-based GV density^26^,^42^ and textural features.^26^,^39^

Machine learning, unlike traditional statistics, is a useful approach when trying to assess a predictive outcome from a large number of input variables.^40^ Several studies have previously been conducted that employ traditional statistical approaches to discriminate subjects with and without femoral neck fractures by measuring high-resolution trabecular architectural variables.^8^,^14^,^35^,^43^ However, there is lack of studies using clinical CT images to elucidate structural risk factors of low-energy acetabular fractures and to discriminate fracture cases from controls using machine learning methods. Hence, this study had two main goals: (1) to investigate whether significant differences can be found between the trabecular architecture of acetabulum and proximal femur in acetabular fracture subjects and their age-gender matched controls; and (2) whether machine learning techniques could discriminate low-energy acetabular fractures based on trabecular architecture and/or proximal femur geometry (PFG).

## Materials and Methods

### Study Subjects and Image Characteristics

The data consisted of abdominopelvic CT images of subjects with acetabular fractures (*n* = 107, 25 females and 82 males) and their age-gender matched controls (*n* = 107).^12^ Clinical images scanned with standard protocols were obtained from the picture archiving and communication system (PACS) of Oulu University Hospital, Oulu, Finland. The images were taken from patients admitted between January 2008 and October 2017. A research permit (220/2017) was obtained from the Northern Ostrobothnia Hospital District, and a written informed consent was not required due to the register-based study design. The exclusion criteria were age (minimum age 50 years), high energy trauma (e.g., car accident), femoral fractures, surgical history, or previous pelvic diseases.

Extraction of volumes of interest (VOI) were taken from two locations: the acetabulum and the femoral head. Initially, there were a total of 214 subjects, 50 females and 164 males. To maintain a quantitative balance between the fracture and control groups, two-sided acetabular fracture cases (14 males) together with their matching controls (14 males) were excluded from the acetabular VOIs. However, these two-sided acetabular fractures were not excluded from the femoral heads VOIs. In addition, 9 images from femoral head VOIs (3 females and 6 males) were eliminated for insufficient quality e.g., structures only party visible.

The final dataset consisted of 186 subjects (*n* = 50 females: mean age ± standard deviation (SD): 77 ± 14 years; *n* = 136 males: 71 ± 11 years) for the acetabular VOIs and 205 subjects (*n* = 47 females: 78 ± 13 years; *n* =158 males: 70 ± 12 years) for femur VOIs. In the final dataset the ratio of cases to controls was 93:93 (females = 25:25, males = 68:68) for acetabular VOIs and 98:107 (females = 22:25, males = 76:82) for femur VOIs. Ages for both genders were normally distributed based on a Shapiro–Wilk test, and females were older on average (*p < 0.05*), the p-value taken by an independent samples *t* test.

CT image properties varied between the fracture and control groups. The average pixel spacing and slice thickness (± SD) were 0.73 ± 0.10 and 1.03 ± 0.68 mm for the fracture group, and 0.77 ± 0.08 and 0.78 ± 0.34 mm for the control group, respectively. As the pixel spacing and slice thickness were different between the groups (*p < 0.05*), we resampled the data to the same voxel sizes (0.8 mm × 0.8 mm × 3mm, see next section).^38^,^44^

### Extraction of Volumes of Interest

**Figure 1:**
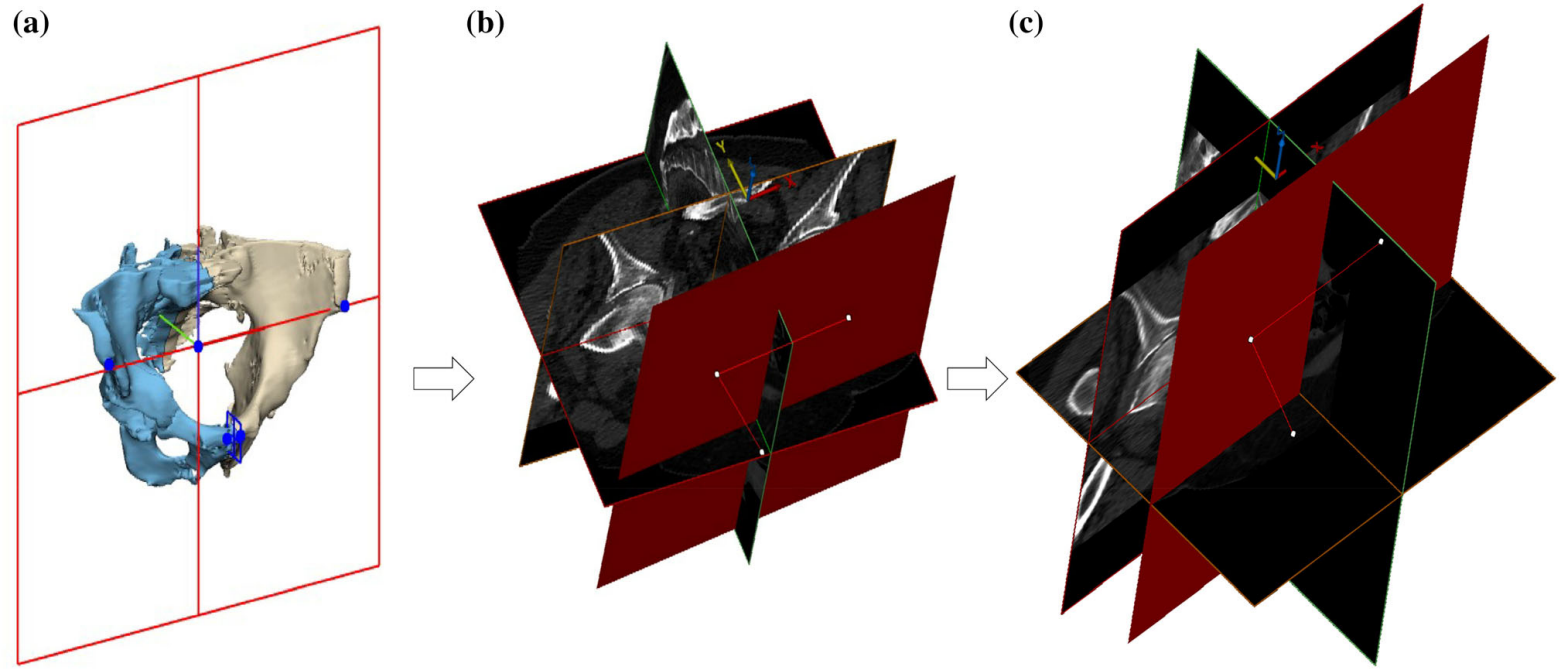
Pelvic slices realignment. (a) shows the creation of a vertical anterior posterior (AP) plane using landmarks on the three-dimensional reconstructed pelvis. (b) shows misalignment between the alignment plane (red) and slices before re-slicing. (c) shows re-aligned slices.

Initially, a 3D reconstruction model of the pelvis was constructed to create an alignment anterior posterior (AP) plane in 3-Matic (Materialise, Leuven BE, Belgium) software.^12^ The plane was formed using ASIS (Anterior Superior Iliac Spine) and PT (Pubic Tubercles) as landmarks and then reoriented parallel to a vertical *XY*-plane. This AP-plane was used as a re-slicing plane in Mimics (Materialise, Leuven BE, Belgium) where the original resolutions were retained (Fig. 1). Each slice was also threshold at a range of − 150 HU (Hounsfield units) to 600 HU and then exported as an 8-bit portable network graphics format image. This range of HU was chosen to ensure that trabecular bone was optimally^5^,^27^ segmented in the selected slices. A custom MATLAB (*version* R2018b, The MathWorks, Inc., Natick, MA, USA) code was written to crop, extract VOIs and to calculate the subsequent variables.

**Figure 2:**
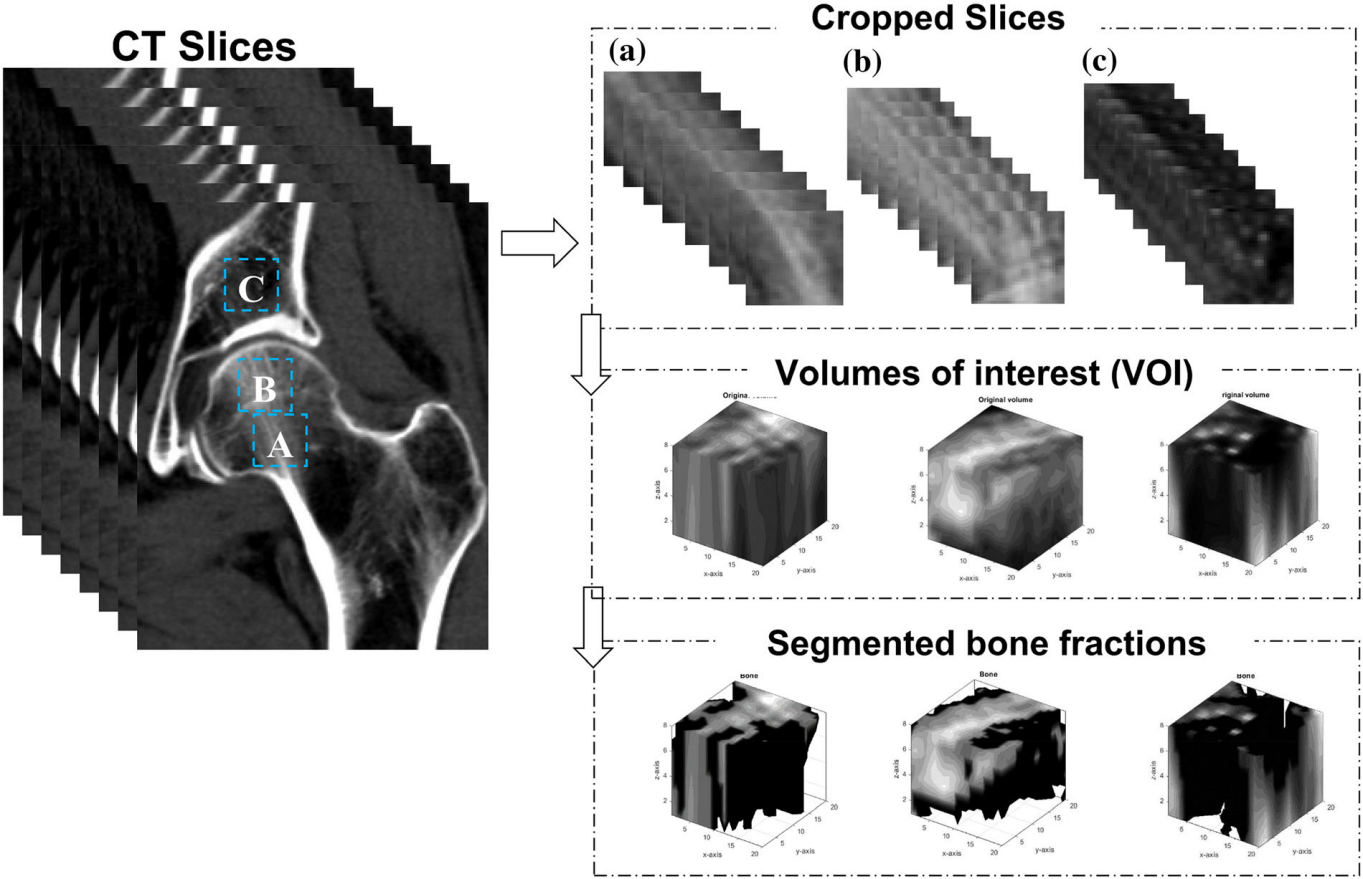
Placement of the volumes of interest (VOI) on the acetabulum and femoral head. (a) and (b) represent femoral head region 1 (FHR-1) and femur head region 2 (FHR-2), respectively, whereas (c) represents the acetabular region (AR). When calculating bone volume fraction, the VOI was segmented into bone fraction and empty space using Otsu thresholding.

A specific number of slices was selected from the realigned CT slices depending on the thickness and depth of VOI (24 mm), i.e., ${\text{number}}\;{\text{of}}\;{\text{slices}} = \left( {{\text{depth}}\;{\text{of}}\;{\text{VOI}}} \right)/\left( {{\text{slice}}\;{\text{thickness}}} \right)$. A rectangular region of interest (ROI = 16 mm × 16 mm) was manually placed in center slice of the selected slices to mark the intended anatomical locations (Fig. 2). Then the ROI was automatically placed on the remaining slices and visually inspected to make sure the demarcated area contained only trabecular bone. Three anatomical locations were selected; the first was on the acetabulum principal compressive unit, i.e., acetabulum region (AR), and the other two were on the femoral head principal compressive unit, i.e., femoral head region FHR-1 and femoral head region FHR-2 (Fig. 2). AR was placed only on the contralateral side of the acetabular fracture, whereas FHR-1 and FHR-2 were placed on both sides both for the fracture and control group.

Then, the area on the CT slices covered by the ROIs was cropped and concatenated to create a VOI $\left( {16 {\text{mm}} \times 16 {\text{mm}} \times 24 {\text{mm}}} \right)$ (Fig. 2). Lastly, each VOI was resampled to the same voxel size $\left( {0.8 {\text{mm}} \times 0.8 {\text{mm}} \times 3 {\text{mm}}} \right)$using bicubic interpolation for comparability of results.^44^ The final dimensions of the VOIs were $\left( {20 \times 20 \times 8} \right) {\text{voxels}}$. The in-plane voxel resolution of 0.8 mm was chosen based on the average pixel spacing of the dataset. The axial voxel resolution of 3 mm was chosen to account for the largest slice-thicknesses in the dataset.

### Proximal Femur Geometry (PFG) Measurement

**Figure 3:**
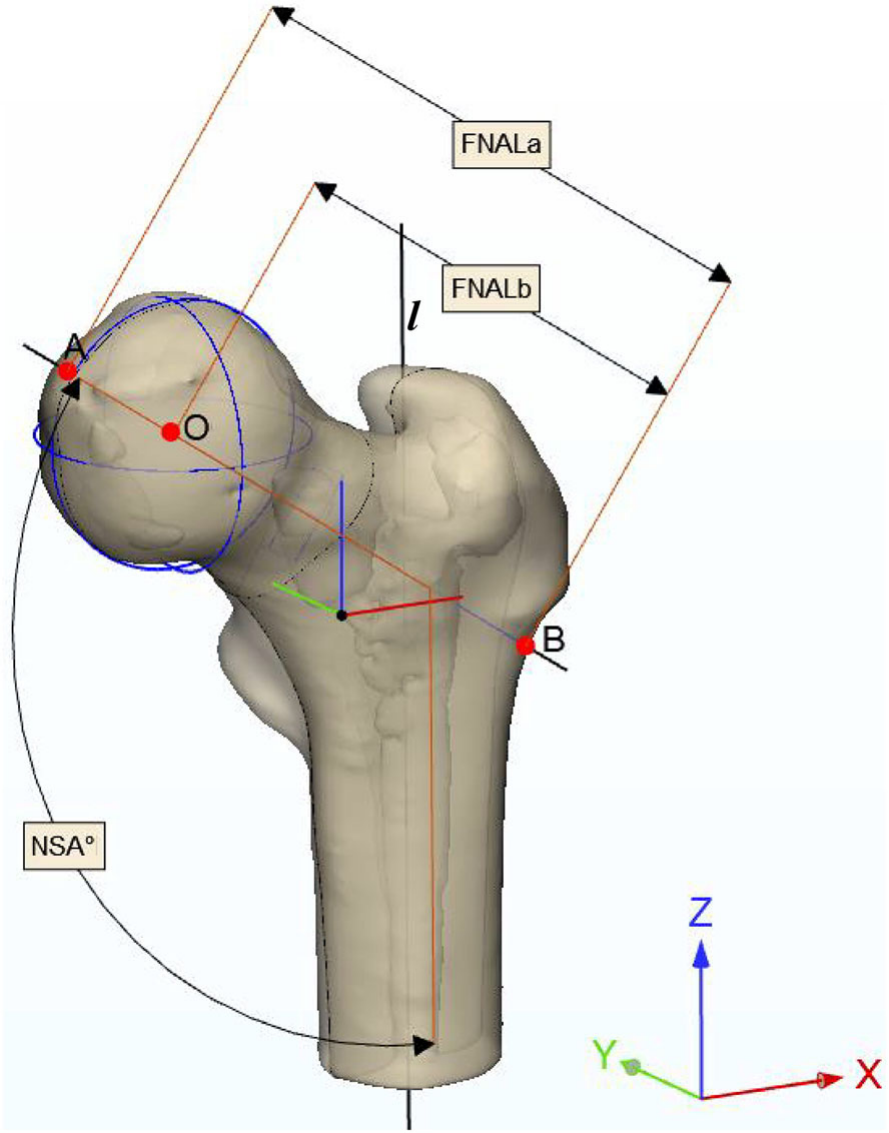
Measurement of Proximal femur geometry (PFG). Neck shaft angle (NSA) is the angle between femoral neck medial axis (A-B) and shaft medial axis (l). (O) is the center of the femoral head, (A-B) is the femoral neck axis length-a (FNALa) and (B-O) is the femoral neck axis length-b (FNALb).

Neck shaft angle (NSA) and femoral neck axis length (FNALa and FNALb) were measured to characterize PFG (Fig. 3). Femurs on the acetabular fracture sides and their corresponding control sides were measured. Briefly, a 3D reconstruction of the femur was created using Mimics and PFG was measured in 3-Matic.^12^ NSA was defined as the angle between the femoral shaft medial axis and the femoral neck medial axis. In addition, the femoral neck length (FNAL) was measured using two parameters,^12^,^34^,^35^ FNALa and FNALb, along the femur neck’s medial axis. The starting point of the medial axis for both variables was below the greater trochanter whereas the FNALa extended up to the femoral head anterior point and FNALb was the distance up to the femoral head center (Fig. 3).

### Bone Density Assessment Using Bone Volume Fraction

BV/TV is a ratio of bone volume (BV) to total volume (TV) which describes the amount of trabecular bone within the boundaries of a VOI.^15^,^21^,^39^ Due to low resolution and lack of a density calibration standard during the acquisition of the CT scans, here BV/TV refers to apparent BV/TV. BV is the total count of bone fraction voxels, while TV is the total number of voxels in the VOI.^31^ Otsu thresholding,^32^ a histogram based adaptive thresholding method, was used to separate BV voxels from TV voxels (Fig. 2).

### Texture Analysis

Texture information of an image or VOI, derived from GLCM, is the spatial distribution of gray levels separated by a given distance at different angles relative to one another.^17^,^18^,^41^ The parameters needed to construct a co-occurrence matrix are *distance*, *offset directions* and *number of gray levels*. Here, 1-pixel distance, 13 offset directions (i.e., the 13 unique angles out of the total of 26 found around a point in 3D) and 16 numbers of gray levels were used. The offset directions were defined by a − 1, 0 or 1 variations of a three-component vector $\left( {\text{row, column, slice}} \right)$that determine the co-occurrence locations of a pair of pixels in the x, y and z Cartesian coordinate system.^39^

The relative frequencies $P_{i,j}$ of two adjacent pixels *i, j* over the 13 offset directions in the VOI were calculated to construct a GLCM of size $\left( {16, 16, 13} \right)$. The $P_{i,j}$ were converted into probabilities by normalizing each by the total number of co-occurrences. In addition, the final GLCM containing the probabilities of co-occurrences was then made symmetrical by adding its transpose along the main diagonal.^18^ Finally, based on these probabilities the following nine variables were calculated; contrast, correlation, entropy, difference entropy, difference variance, homogeneity, maximum probability, sum variance and information measure of correlation.^17^,^18^,^41^ Briefly, contrast, correlation, entropy and homogeneity describe local intensity variations, linear dependencies, disorder and similarities between neighboring gray levels, respectively.^18^,^41^ Difference entropy and difference variance measure the disorder related to gray level differences and heterogeneity with respect to the mean respectively.^18^ Maximum probability describes the largest probability of a gray level’s co-occurrence in the GLCM.^17^ Sum variance describes the sum distribution of the gray level around the GLCM mean.^18^,^41^ Information measure of correlation (IMC) is a measure of texture complexity derived from mutual information as defined by Haralick *et al*.^18^ Lastly, the variables in each of the 13 directions were averaged to ensure rotational invariance.^26^,^39^

### Bone Density Assessment Using Gray Value Histogram Analyses

Histogram analyses are first order statistics that provide information about gray value (GV) distributions within an image or VOI. Previously, histograms have been used to indirectly assess trabecular bone density.^22^,^26^,^42^ GV mean, standard deviation, variance, skewness and kurtosis were calculated in this study.

### Statistical Analyses

Prior to mean group comparisons, a Shapiro–Wilk test was performed to determine variable distribution. Following the normality check, either an independent-samples *t* test or a Mann–Whitney *U* test was conducted for normally or nonnormally distributed data, respectively. Correction for multiple comparison testing was not applied.^37^ A bivariate Pearson’s linear correlation was also performed to investigate the associations between variables and to check for multicollinearity. The IBM SPSS (*version* 24.0.0.1, Armonk, CINY: IBM Corp, USA) statistics program was used for statistical analyses.

Two machine learning methods, Bayesian logistic regression (BLR) and Elastic net (EN) models, were implemented to discriminate acetabular fracture cases from controls. The models classified the responses into the pair “fracture” and “control” for FHR-1 and FHR-2, comparing the fracture side of the acetabular fracture cases and the matching side of the controls, and into the pair “contralateral” and “control-contralateral*”* for AR, comparing the contralateral side of the fracture cases, and the matching contralateral side of the controls, respectively. In addition, classification performances of the models were evaluated using the area under the receiver operating characteristics (ROC) curve (AUC).

To identify the best predictive model and for easy interpretability of the classification results, three types of model inputs were used; PFG alone, and trabecular variables without and with PFG. Inputs without PFG comprised of BV/TV, GLCM texture and GV histogram variables, and those with PFG consisted of the additional geometry variables of the proximal femur. In order to train and validate the models, repeated *k*-fold cross-validation (CV) was used at 10 folds and 50 repeats. With K-fold CV data is split into equal sized training sections and validated iteratively on a random *k*th part *k* number of times.^20^ R (*version* 3.6.1) statistical computing software was used for the machine learning analyses. We used the R package caret^28^ (*version* 6.0-84) for the k-fold CV, arm^13^ (*version* 1.10-1) for BLR, glmnet^11^ (*version* 3.0) for EN models and pROC^36^ (version 1.15.3) to plot the ROC curves.

#### Bayesian Logistic Regression (BLR)

BLR is a type of regression developed as an improvement to the traditional logistic regression. It mainly solves nonidentifiability and unstable separation problems that are especially associated with smaller datasets.^13^ In order to accomplish this, it standardizes both the binary predictors and continuous input variables and then assigns independent samples *t* test priors on the regression coefficients $\beta$ 13

Principal component analysis (PCA) was performed before running BLR to account for multicollinearity between the input variables. Here, PCA was used for dimension reduction by decomposing the data into orthogonal principal components (PCs) in the direction of maximum variance. Eigenvectors of the covariance matrix were calculated to determine the PCs’ variances. PCs that explained ≥ 98% of the variance were finally chosen as model inputs. For instance, for the two input types, without and with PFG, there were 15 and 18 variables which were reduced to 8 and 10 PCs respectively after the PCA.

#### Elastic Net (EN) Regression

EN is a type of regularized linear regression used in statistics for shrinking coefficients in order to reduce the effects of multicollinearity and optimize feature selection.^45^ It is particularly useful for when the number of observations *N* is less than the number of predictor variables $x$.^45^ EN is a combination of two penalties, the lasso (*L*_1_, *α* = 1) and ridge regression (*L*_2_, *α* = 0), incorporated into the standard linear regression to reduce $\beta$ to zero, given the response variable $y$ and the regularization parameter *λ* (Eq. 1).^45^ The *λ* parameter is inversely related to the number of penalized nonzero $\beta$ values and hence determines the strength of penalty.^45^

1 $$\mathop {\hbox{min} }\limits_{{\beta_{0} ,\beta }} \left( {\frac{1}{2N}\mathop \sum \limits_{i = 1}^{N} \left( {y_{i} - \beta_{0} - x_{i}^{T} \beta } \right)^{2} + \lambda \left[ {\frac{{\left( {1 - \alpha } \right)}}{2}\mathop \sum \limits_{j = 1}^{p} \beta_{j}^{2} + \alpha \mathop \sum \limits_{j = 1}^{p} \left| {\beta_{j} } \right|} \right]} \right)$$

Each *k*-fold CV model was fit repeatedly over a grid of regularization parameters (*α*, *λ*) to solve the EN regression problem (Eq. 1). The grid search consisted of α ranging from 0.01 to 1 incremented at 0.03 and *λ* from 0.001 to 0.15 incremented at 0.005.

## Results

When comparing the trabecular architecture between the fracture subjects and their matching control sides at the acetabula and femoral heads, the variables which showed significant differences (*p < 0.05*) varied amongst the three VOIs (Tables 1, 2, and 3). Moreover, a strong multicollinearity within the GLCM texture and GV histogram variables was observed for all three VOIs (Supplementary Tables 1–3).

**Table 1:** Bone volume fraction (BV/TV), gray level co-occurrence matrix and gray value (GV) histogram variables of the acetabular region (AR) measured on the contralateral side of subjects with acetabular fracture and the matching side of the non-fractured controls.

Variables	Acetabular fracture contralateral side			Controls contralateral side			*p*		
	All (*n* = 93)	F (*n* = 25)	M (*n* = 68)	All (*n* = 93)	F (*n*=25)	M (*n* = 68)	All (*n* = 186)	F (*n* = 50)	M (*n* = 136)
BV/TV	0.26 (0.13)	0.24 (0.13)	0.27 (0.93)	0.26 (0.13)	0.23 (0.14)	0.27 (0.93)	0.807	0.745	0.932
Contrast	2.92 (1.26)	2.69 (1.22)	3.01 (1.27)	3.25 (1.29)	3.44 (1.19)	3.18 (1.33)	0.054	0.024^b^	0.426
Homogeneity	0.60 (0.06)	0.62 (0.06)	0.60 (0.05)	0.60 (0.05)	0.60 (0.06)	0.59 (0.05)	0.308	0.300	0.605
Correlation	0.71 (0.08)	0.71 (0.1)	0.71 (0.08)	0.74 (0.05)	0.72 (0.06)	0.74 (0.05)	0.016^b^	0.915	0.006^b^
Entropy	3.69 (0.42)	3.54 (0.47)	3.74 (0.39)	3.83 (0.43)	3.78 (0.48)	3.85 (0.41)	0.020^a^	0.092	0.098
Difference entropy	1.34 (0.17)	1.3 (0.18)	1.36 (0.16)	1.38 (0.16)	1.39 (0.17)	1.38 (0.16)	0.089	0.072	0.397
Difference variance	1.46 (0.59)	1.39 (0.61)	1.48 (0.59)	1.65 (0.67)	1.86 (0.63)	1.58 (0.67)	0.041^b^	0.008^b^	0.486
Sum variance	18.35 (8.86)	17.25 (9.36)	18.76 (8.7)	22.57 (10.21)	22.73 (11.14)	22.51 (9.93)	0.002^b^	0.070	0.014^b^
Maximum probability	0.10 (0.04)	0.11 (0.05)	0.09 (0.04)	0.09 (0.04)	0.09 (0.04)	0.09 (0.04)	0.081	0.146	0.221
IMC	− 0.21 (0.05)	− 0.21 (0.05)	− 0.20 (0.05)	− 0.22 (0.04)	− 0.22 (0.03)	− 0.22 (0.04)	0.156	0.624	0.069
GV mean	98.81 (29.04)	90.88 (36.58)	101.73 (25.43)	115.33 (26.7)	110.02 (31.92)	117.28 (24.49)	< 0.001^a^	0.035^b^	< 0.001^a^
GV SD	34.73 (8.57)	35 (7.98)	34.63 (8.83)	37.46 (8.35)	37.66 (8.81)	37.39 (8.24)	0.029^a^	0.268	0.062
GV variance	1.28 (0.62)	1.28 (0.56)	1.28 (0.64)	1.47 (0.66)	1.49 (0.68)	1.46 (0.65)	0.038^b^	0.253	0.073^b^
GV skewness	1.37 (0.7)	1.63 (0.82)	1.28 (0.63)	1.27 (0.71)	1.45 (0.85)	1.21 (0.65)	0.153	0.450	0.284
GV kurtosis	6.73 (3.39)	8.16 (4.36)	6.21 (2.81)	5.89 (3.39)	6.62 (4.03)	5.62 (3.12)	0.036^b^	0.138	0.128

Values are given as mean and standard deviation (SD). Statistical *p* values of the differences for all subjects and individual genders are also shown

*IMC* information measure of correlation

^a^Parametric Independent samples *t* test (*p < 0.05*)

^b^Nonparametric Mann–Whitney *U* test (*p < 0.05*)

**Table 2:** Bone volume fraction (BV/TV), gray level co-occurrence matrix and gray value (GV) histogram variables of the femoral head region 1 (FHR-1) measured on the fracture side of subjects with acetabular fracture and the matching side of the non-fractured controls.

Variables	Fracture side			Control side			*p*		
	All (*n* = 98)	F (*n* = 22)	M (*n* = 76)	All (*n* = 107)	F (*n* = 25)	M (*n* = 82)	All (*n* = 205)	F (*n* = 47)	M (*n* = 158)
BV/TV	0.49 (0.09)	0.45 (0.07)	0.49 (0.08)	0.50 (0.09)	0.46 (0.07)	0.52 (0.08)	0.182	0.856	0.141
Contrast	3.45 (1.23)	3.54 (1.28)	3.48 (1.22)	4.02 (1.35)	3.76 (1.313)	4.10 (1.35)	0.001^b^	0.815	< 0.001^b^
Homogeneity	0.56 (0.04)	0.55 (0.04)	0.56 (0.04)	0.55 (0.03)	0.55 (0.03)	0.55 (0.03)	0.038^b^	0.991	0.002^b^
Correlation	0.77 (0.07)	0.77 (0.07)	0.77 (0.07)	0.78 (0.06)	0.78 (0.05)	0.78 (0.07)	0.548	0.815	0.566
Entropy	1.42 (0.13)	1.44 (0.14)	1.41 (0.14)	1.47 (0.12)	1.45 (0.13)	1.47 (0.12)	0.003^b^	0.949	0.002^a^
Difference entropy	1.43 (0.52)	1.49 (0.516)	1.41 (0.53)	1.68 (0.6)	1.59 (0.58)	1.71 (0.60)	< 0.001^b^	0.685	< 0.001^b^
Difference variance	4.18 (0.26)	4.24 (0.22)	4.17 (0.27)	4.29 (0.23)	4.25 (0.23)	4.29 (0.23)	0.002^a^	0.898	0.001^a^
Sum variance	28.77 (10.34)	28.68 (7.98)	28.79 (10.98)	34.52 (12.29)	31.72 (8.84)	35.37 (13.09)	< 0.001^b^	0.225	0.001^b^
Maximum probability	0.05 (0.02)	0.04 (0.02)	0.05 (0.02)	0.05 (0.03)	0.05 (0.02)	0.05 (0.03)	0.145	0.224	0.022^b^
IMC	− 0.24 (0.05)	− 0.24 (0.05)	− 0.25 (0.05)	− 0.25 (0.04)	− 0.24 (0.03)	− 0.25 (0.04)	0.641	0.915	0.681
GV mean	136.41 (27.5)	127.61 (29.05)	138.96 (26.69)	147.99 (27.85)	134.51 (35.02)	152.10 (24.05)	0.003^a^	0.470	0.001^a^
GV SD	40.68 (9.58)	42.35 (6.84)	40.19 (10.23)	44. (9.2)	42.39 (8.51)	44.49 (9.39)	0.012^a^	0.987	0.007^b^
GV variance	1.74 (0.82)	1.84 (0.57)	1.72 (0.87)	2.02 (0.85)	1.87 (0.71)	2.07 (0.89)	0.016^b^	0.883	0.007^b^
GV skewness	0.08 (0.37)	0.19 (0.25)	0.04 (0.39)	0.01 (0.39)	0.21 (0.37)	− 0.05 (0.37)	0.208	0.859	0.123
GV kurtosis	2.71 (0.62)	2.61 (0.41)	2.74 (0.67)	2.49 (0.5)	2.57 (0.43)	2.47 (0.51)	0.003^b^	0.509	0.003^b^

Values are given as mean and standard deviation (SD). Statistical test *p* values of the differences for all subjects and individual genders are also shown

*IMC* information measure of correlation

^a^Parametric Independent samples *t* test (*p < 0.05*)

^b^Nonparametric Mann–Whitney *U* test (*p < 0.05*)

**Table 3:** Bone volume fraction (BV/TV), gray level co-occurrence matrix and gray value (GV) histogram variables of the femoral head region 2 (FHR-2) measured on the fracture side of subjects with acetabular fracture and the matching side of the non-fractured controls.

Variables	Fracture side			Control side			*p*		
	All (*n* = 98)	F (*n* = 22)	M (*n* = 76)	All (*n* = 107)	F (*n* = 25)	M (*n* = 82)	All (*n* = 205)	F (*n* = 47)	M (*n* = 158)
BV/TV	0.51 (0.12)	0.49 (0.09)	0.52 (0.12)	0.55 (0.11)	0.49 (0.09)	0.56 (0.11)	0.012^a^	0.829	0.011^a^
Contrast	2.97 (1.18)	3.07 (1.70)	2.94 (1.00)	3.17 (1.30)	2.76 (0.85)	3.29 (1.39)	0.130	0.701	0.125
Homogeneity	0.58 (0.04)	0.58 (0.05)	0.58 (0.04)	0.58 (0.05)	0.59 (0.03)	0.58 (0.05)	0.817	0.565	0.609
Correlation	0.77 (0.08)	0.76 (0.08)	0.77 (0.08)	0.79 (0.06)	0.80 (0.049)	0.79 (0.06)	0.004^b^	0.024^a^	0.024^b^
Entropy	1.37 (0.14)	1.37 (0.17)	1.36 (0.13)	1.37 (0.14)	1.34 (0.13)	1.39 (0.14)	0.419	0.966	0.310
Difference entropy	1.37 (0.51)	1.33 (0.67)	1.33 (0.46)	1.44 (0.59)	1.25 (0.38)	1.50 (0.63)	0.081	0.733	0.081
Difference variance	4.03 (0.28)	4.05 (0.31)	4.02 (0.26)	4.09 (0.36)	4.05 (0.29)	4.10 (0.38)	0.011^b^	0.988	0.006^b^
Sum variance	23.80 (8.75)	22.62 (7.26)	24.14 (9.15)	29.26 (10.44)	26.94 (9.38)	29.96 (10.69)	< 0.001^b^	0.088	< 0.001^b^
Maximum probability	0.07 (0.03)	0.06 (0.02)	0.07 (0.03)	0.07 (0.08)	0.07 (0.03)	0.07 (0.08)	0.063	0.339	0.017^b^
IMC	− 0.24 (0.06)	− 0.23 (0.05)	− 0.25 (0.06)	− 0.27 (0.04)	− 0.27 (0.04)	− 0.26 (0.04)	0.001^b^	0.011^a^	0.018^a^
GV mean	150.63 (31.54)	137.43 (29.71)	154.45 (31.2)	163.47 (32.67)	142.5 (39.28)	169.86 (27.61)	0.005^†^	0.624	0.001^a^
GV SD	33.93 (8.15)	33.3 (6.70)	34.11 (8.55)	38.16 (8.48)	34.91 (6.66)	39.15 (8.76)	< 0.001^†^	0.416	< 0.001^a^
GV variance	1.22 (0.58)	1.15 (0.47)	1.24 (0.62)	1.53 (0.69)	1.26 (0.47)	1.61 (0.73)	0.001^b^	0.394	0.001^b^
GV skewness	0.01 (0.49)	0.15 (0.35)	− 0.03 (0.52)	− 0.18 (0.57)	0.11 (0.45)	− 0.27 (0.57)	0.023^b^	0.624	0.022^b^
GV kurtosis	3.23 (0.76)	3.19 (0.75)	3.24 (0.77)	3.04 (1.21)	2.91 (0.59)	3.07 (1.34)	0.002^b^	0.159	0.007^b^

Values are given as mean and standard deviation (SD). Statistical test *p* values of the differences for all subjects and individual genders are also shown

IMC = information measure of correlation

^a^Parametric Independent samples *t* test (*p < 0.05*)

^b^Nonparametric Mann–Whitney *U* test (*p < 0.05*)

### Trabecular Architecture at Acetabulum

There were no significant differences when comparing the BV/TV at AR on the contralateral side of acetabular fracture subjects with their matching contralateral sides of control subjects. Amongst the GLCM texture variables at AR, entropy was significantly smaller on the contralateral sides of the fractured subjects than on the matching contralateral sides of the controls only for all subjects (3.69 [95% Confidence Interval (CI)] [3.60–3.77] vs. 3.83 [3.74–3.92], *p = 0.02*) (Table 1). In addition, correlation and sum variance for all subjects and males, and contrast and difference variance for all subjects and females were significantly lower on the subjects with acetabular fracture compared to their matching controls (*p < 0.05*) (Table 1).

Amongst the AR GV histogram variables, GV mean was significantly lower on the contralateral sides of the fracture subjects than their matching controls for all subjects (98.81 [92.83–104.79] vs. 115.33 [109.83–120.83], *p < 0.001*), females (90.88 [75.78–105.98] vs. 110.02 [96.84–123.19], *p = 0.035*) and males (101.73 [95.57–107.88] vs. 117.28 [117.28–111.35], *p < 0.001*) (Table 1).

### Trabecular Architecture at Femoral Head

None of the textural variables at FHR-1 for female subjects showed significant differences between the femoral heads of the fracture side of the subjects with acetabular fracture and the matching side of the non-fractured controls (Table 2). The homogeneity at FHR-1 was greater on the fracture sides than controls for all subjects (0.56 [0.55–0.57] vs. 0.55 [0.54–0.55], *p = 0.038*) and males (0.56 [0.55–0.57]–0.55 [0.54–0.55], *p = 0.002*). Also, FHR-2 of females showed differences only for correlation and IMC (Table 3).

BV/TV at FHR-1 did not show difference between the groups (Table 2). However, BV/TV at FHR-2 was significantly lower on the fracture sides than on the matching control sides for all subjects (0.51 [0.48–0.53] vs. 0.55 [0.53–0.57], *p = 0.012*) and males (0.52 [0.49–0.54] vs. 0.56 [0.54–0.59], *p = 0.011*) (Table 3).

Amongst the GV histogram variables at FHR-1, GV mean was significantly lower on the fracture sides than on the matching control sides for all subjects (136.41 [130.90–141.93] vs. 147.99 [142.65–153.32], *p = 0.003*) and males (138.96 [132.86–145.06] vs. 152.10 [146.81–157.38], *p = 0.001*) (Table 2). Similarly, at FHR-2, the GV mean was significantly lower on the fracture sides than on the matching control sides for all subjects (150.63 [144.31–156.96] vs. 163.47 [157.21–169.73], *p = 0.005*) and males (154.45 [147.32–161.58] vs. 169.86 [163.79–175.93], *p = 0.001*) (Table 3). In addition, the GV skewness did not show difference at FHR-1 (Table 2) while at FHR-2 it was negatively skewed for the control cases for all subjects (0.01 [− 0.08 to 0.11] vs. − 0.18 [− 0.29 to − 0.07], *p = 0.023*) and males (− 0.03 [− 0.15 to 0.09] vs. − 0.27 [− 0.39 to − 0.14], *p = 0.022*) (Table 3).

Lastly, the side-wise relationships of the trabecular variables between FHR-1 and FHR-2 on their respective fracture and control sides are presented in the Supplementary Table 4. Strong correlation was found between most variables.

### Proximal Femur Geometry (PFG)

Acetabular fracture subjects had significantly smaller NSA than controls for all subjects (121.71° [120.58°–122.84°] vs. 124.60° [123.49°–125.70°], *p < 0.001*), for females (118.76° [115.72°–121.79°] vs. 124.51° [122.09°–126.93°], *p = 0.003*) and also for males (122.49° [121.33°–123.66°] vs. 124.63° [123.36°–125.89°], *p = 0.015*). In contrast, FNALb was significantly longer for fractures than controls for all subjects (78.36 mm [77.14–79.58 mm] vs. 76.03 mm [74.75–77.30 mm], *p = 0.010*) and males (80.07 mm [78.87–81.28 mm] vs. 77.77 mm [76.42–79.12 mm], *p = 0.037*) (Supplementary Table 5).

### Classification Performance

**Figure 4:**
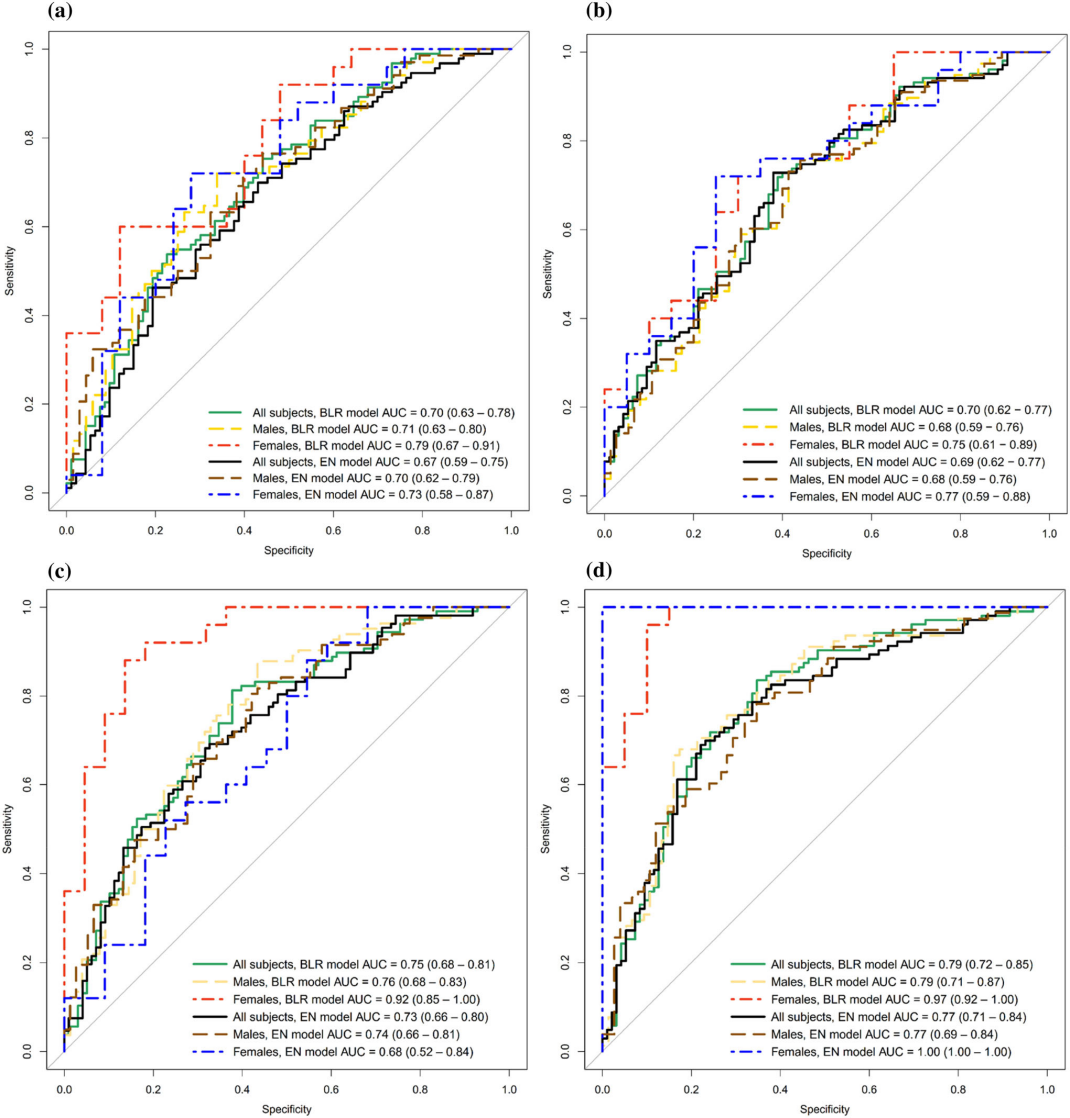
Receiver operating characteristics curves based on inputs from the acetabular region (AR), the combined femoral head regions (FHR) 1 and 2, and proximal femur geometry (PFG). Bayesian logistic regression (BLR) and elastic net (EN) models were applied for all subjects and individual genders. FHR and AR were measured from the fracture and contralateral side, respectively. (a) shows AR curves, (b) shows PFG curves, (c) and (d) show combined FHR curves, without and with PFG.

When assessing the classification performances of the AR BLR and EN models, the ROC AUC values were 0.70 [0.63–0.78] and 0.68 [0.60–0.76] for all subjects, 0.88 [0.78–0.98] and 0.86 [0.76–0.97] for females, 0.72 [0.63–0.81] and 0.69 [0.60–0.79] for males, respectively (Table 4 and Fig. 4). The variables selected in the final AR EN models are shown in Supplementary Table 6.

**Table 4:** The area under the curve (AUC) values in the ROC analysis for the classification performances of the Bayesian logistic regression (BLR) and elastic net (EN) models.

Model Inputs	BLR AUC (95% CI)			EN AUC (95% CI)		
	All	Females	Males	All	Females	Males
PFG						
Fracture side	0.70 (0.62–0.77)	0.75 (0.61–0.89)	0.68 (0.59–0.76)	0.69 (0.62–0.77)	0.74 (0.59–0.89)	0.68 (0.59–0.76)
Trabecular structure, density and texture variables						
AR	0.70 (0.63–0.78)	0.79 (0.67–0.91)	0.71 (0.63–0.80)	0.67 (0.59–0.75)	0.73 (0.58–0.87)	0.70 (0.62–0.79)
FHR-1	0.70 (0.63–0.77)	0.69 (0.53–0.85)	0.72 (0.65–0.80)	0.70 (0.63–0.77)	0.82 (0.70–0.95)	0.70 (0.62–0.78)
FHR-2	0.72 (0.65–0.79)	0.78 (0.65–0.91)	0.73 (0.66–0.81)	0.70 (0.63–0.77)	0.77 (0.63–0.91)	0.72 (0.64–0.80)
FHR-1&2	0.75 (0.68–0.81)	0.92 (0.85–1.00)	0.76 (0.68–0.83)	0.73 (0.66–0.80)	0.68 (0.52–0.84)	0.74 (0.66–0.81)
Trabecular structure, density and texture variables + PFG						
AR	–	–	–	–	–	–
FHR-1	0.76 (0.70–0.83)	0.83 (0.72–0.95)	0.77 (0.70–0.84)	0.76 (0.70–0.83)	0.74 (0.59–0.89)	0.77 (0.70–0.85)
FHR-2	0.76 (0.70–0.83)	0.93 (0.86–1.00)	0.77 (0.69–0.85)	0.76 (0.69–0.83)	0.92 (0.83–1.00)	0.76 (0.69–0.84)
FHR-1&2	0.79 (0.72–0.85)	0.97 (0.92–1.00)	0.79 (0.71–0.87)	0.77 (0.71–0.84)	1.00 (1.00–1.00)	0.77 (0.69–0.84)

The fracture side proximal femur geometry (PFG) was used in the femoral head region (FHR) models

When assessing the classification performances of the PFG BLR and EN models, the ROC AUC values were 0.70 [0.62–0.77] and 0.69 [0.62–0.77] for all subjects, 0.75 [0.61–0.89] and 0.74 [0.59–0.89] for females, 0.68 [0.59–0.76] and 0.68 [0.59–0.76] for males, respectively (Table 4 and Fig. 4).

Without the inclusion of PFG, the highest BLR and EN ROC AUC values for FHR-1 and FHR-2 variables were 0.72 for all subjects and 0.82 for the individual genders (Table 4). When FHR-1 and FHR-2 variables were combined into one input, the BLR and EN ROC AUCs were 0.75 [0.68–0.81] and 0.73 [0.66–0.80] for all subjects, 0.92 [0.85–1.00] and 0.68 [0.52–0.84] for females, 0.76 [0.68–0.83] and 0.74 [0.66–0.81] for males, respectively. With the inclusion of PFG, the BLR and EN ROC AUCs for the combined FHR-1 and FHR-2 variables were 0.79 [0.72–0.85] and 0.77 [0.71–0.84] for all subjects, 0.97 [0.92–1.00] and 1.00 [1.00–1.00] for females, 0.79 [0.71–0.88] and 0.77 [0.69–0.84] for males, respectively (Table 4 and Fig. 4). The variables selected in the final EN model of the combined features of FHR-1, FHR-2 and PFG are shown in Table 5.

**Table 5:** Coefficient weights of the variables used in the final EN model of the combined inputs of trabecular architecture features at the femoral head region -1 (FHR-1) and femoral head region -2 (FHR-2), and proximal femur geometry (PFG) defined by neck shaft angle(NSA) and femoral neck axis length (FNALa and FNALb).

Variables	Weights
Intercept	0.092
Difference entropy FHR-1	0.186
Difference entropy FHR-2	0.100
Entropy FHR-1	0.110
Sum variance FHR-2	0.015
IMC FHR-2	− 0.445
GV mean FHR-1	0.107
GV mean FHR-2	0.108
GV kurtosis FHR-1	− 0.096
NSA	0.365
FNALb	− 0.322

The trabecular architecture features were bone volume fracture (BV/TV), gray level co-occurrence matrix and gray value (GV) histogram variables

*IMC* information measure of correlation

The regularization parameters used in the final EN models are shown in Supplementary Table 7.

## Discussion

In this study, trabecular architecture of acetabulum and femoral head as well as proximal femur geometry were measured on clinical CT images to identify potential structural risk factors of acetabular fractures. Discrimination of acetabular fracture cases from controls was also implemented using machine learning methods. We found lower trabecular bone volume fraction at the femoral head region close to the hip joint (FHR-2) and lower density (histogram-based variables) at both the acetabulum and femoral head of the fracture subjects compared to their matched controls. Furthermore, we observed difference in the trabecular architecture between the femoral heads of the fracture and control subjects. For the first time, we showed that trabecular architecture as well as proximal femur geometry, both alone and when combined, are able to discriminate acetabular fracture cases from controls. The highest discriminative capacity was observed for the combination of femoral head trabecular architecture and PFG variables (AUC 0.77 to 0.79).

We found BV/TV to be 0.50 ± 0.09, 0.55 ± 0.11 and 0.26 ± 0.13 respectively at FHR-1, FHR-2 and AR for our non-fracture control subjects. We did not observe any BV/TV differences at the femoral head region FHR-1 between the fractures and controls suggesting normal trabecular bone volume. Thevenot *et al*.^42^ extracted VOIs at the femoral head-neck region close to our FHR-1, and reported BV/TV as 0.48 ± 0.27, which closely matches to our findings. For the FHR-2 region, BV/TV of the fracture subjects, especially males, was significantly different from that of the matching sides of controls, indicating that the trabeculae at the femur head might have abnormal or different structure in the cases with acetabular fracture. BV/TV at the acetabular region was not significantly different between the contralateral side of the fractured cases and matching side of the controls. However, the acetabulum of the fractured side was not assessed since a fracture within the region could yield unreliable results.

To define texture, we calculated nine GLCM based statistical variables, as defined by Haralick *et al*.^17^,^18^ When analyzing textural differences between controls and fracture cases, the matched side comparisons revealed differences for all the three VOIs. For the AR, only slight textural differences were observed, with the females showing significantly fewer local variations in the gray levels and males demonstrated significantly lesser linear dependence and dispersion of gray level sums from the mean at the contralateral sides. In addition, a general linear model (GLM) univariate analysis was performed to further understand the independent contribution of texture variables by controlling for the effects of GV (data not shown). After adjusting with the GV mean, Entropy, Difference variance, and Sum variance did not significantly differ between controls and fracture cases anymore. Conversely, IMC showed significant difference between controls and fracture cases after adjustment. It should be noted here that we were unable to find previous literature on GLCM based texture analysis at the AR.

Previous GLCM texture studies of the proximal femur placed VOIs at different locations than this study,^35^,^43^ hence it is difficult to make an assertive comparisons to our findings. The texture at FHR-1 was significantly more homogeneous and less entropic for fracture subjects consistent with previous reporting for femoral neck fracture studies.^35^,^43^ On the contrary, FHR-2 gray levels had similar homogeneity and disorder between the fracture and control subjects. After adjusting with the GV mean, homogeneity in FHR-1 and Difference Variance in FHR-2 did not significantly differ between controls and fracture cases anymore. In addition, the side-wise comparisons of the femoral head VOIs revealed that FHR-2 is significantly more homogeneous and less entropic, with gray levels deviating less than within FHR-1, suggesting that these two VOIs have different textures. However, information measure of correlation (IMC) showed different results for the side-wise comparison of the femoral head VOIs. The acetabular fracture side femoral head IMC comparison did not show a significant difference between the two VOIs, which could be due to the similarity of the texture complexities, while FHR-2 showed a significantly different texture complexity than FHR-1 at the control side.

Presently, GV mean was used as a measure of density^23^,^26^,^42^ and the results of the fracture- vs. -control comparisons suggest that fracture subjects have lesser dense trabecular bone at all of the three regions. Here, we did not get a strong correlation between GV mean and BV/TV at the AR, but we did get moderate correlations (*r* > 0.55, *p < 0.01*) within the femoral head. Previous µCT to clinical CT co-registered studies have reported different values of correlation between GV mean and BV/TV (*r* = 0.91, *p < 0.01*,^26^ and *r* = 0.61, *p < 0.01*).^23^ The correlation differences in these studies might be due to the variations in resolution, anatomical locations and/or methodologies used to calculate GVs.^23^ Furthermore, the GV skewness suggests tendency of the individual GV mean distribution differences within the fracture and control groups. For the control subjects, the GV histogram of FHR-2 was significantly negatively skewed compared to fracture subjects, suggesting FHR-2 as much denser only for the controls. The side-wise comparisons for males and combined gender subjects on the fracture and control sides revealed FHR-2 to be denser than FHR-1. However, the skewness results on the fracture side suggest similar density distributions between FHR-1 and FHR-2, but on the control side a significantly negatively skewed FHR-2 GV mean distribution.

A possible explanation for an acetabular fracture as a result of a low energy sideway impact could be PFG, femoral head trabeculae connectivity and/or anisotropy. Our current results show smaller NSA (121.7° vs. 124.6°) and lower density at the femoral head in the acetabular fracture subjects. Normal femoral head trabeculae are plate-like^21^ but due to osteoporosis they become more rod-like with a loss of horizontal connectivity and increased anisotropy.^2^ Trabecular architecture is subject to age and gender-related changes.^2^,^3^,^8^ In addition, our results of BV/TV and GLCM texture indicate that FHR-2 for fracture subjects is structurally abnormal, and also more similar to FHR-1 in structure and complexity of texture. Therefore, PFG differences in combination with an abnormal trabecular architecture, possibly due to the loss of horizontal connectivity and/or variations in anisotropy, may affect the hip joint stress/strain distribution following impact thence causing acetabular fracture(s).

For the first time, we presented the application of Bayesian logistic regression and Elastic net machine learning methods to classify low-energy acetabular fracture subjects from their age-gender matched controls using trabecular architectural variables with and without the inclusion of PFG. Two different types of machine learning approaches were implemented to compare classification performances where multicollinearity of the model inputs was handled differently in each case. In our previous 3D pelvic geometry study, we have shown that a Varus femur with longer FNALb (> 78mm) could be associated with acetabular fractures.^12^ In the current study, the PFG BLR and EN machine learning models were able to discriminate acetabular fractures from controls (AUC 0.68–0.75). The current findings are in-line with prior 2D radiographic studies that have shown NSA to discriminate femoral neck fractures from non-fracture femurs, AUC being 0.72 (Gnudi *et al*.^14^), 0.69 (Thevenot *et al*.^43^) and 0.87 (Pulkkinen *et al*.^35^).

The different types of variables used in the final EN model show that the discrimination of acetabular fractures from controls is best achieved by combining trabecular architecture and PFG. The machine learning models with the highest classification performances were found when FHR-1, FHR-2 and PFG variables were combined (AUC > 0.77). Some of the selected variables used in the final EN model were GV mean at FHR-1 and FHR-2 from the GV histogram features, IMC at FHR-2, difference entropy and entropy at FHR-1 from the 3D GLCM texture features, and from PFG NSA and FNALb (Table 5). Therefore, by using the BLR and EN machine learning methods we were able to regularize a relatively large number of inputs with a high degree of multicollinearity to identify the most important variables to discriminate acetabular fractures from controls.

This study has some limitations. Firstly, the CT data was collected from clinical setting, where the patient positioning was not standardized, and a density calibration phantom was not used. Hence, all CT slices were aligned to a vertical plane to standardize the pelvic orientation before VOIs were placed at their designated anatomical locations. In addition, due to the absence of a density calibration phantom bone mineral density matching was not possible. Secondly, because of the low-resolution detailed trabecular microstructure measurements were not possible. However, texture analyses provided information relevant to microstructure.^6^,^39^ Thirdly, the sample size for females was limited as was evident in variation in AUC suggesting possible under-or over-estimations. In addition, we were not able to perform trabecular analysis of the fractured acetabulum and used the contralateral side for the analysis. Further studies are needed for final confirmation of the findings.

In conclusion, we were able to discriminate acetabular fractures from controls using clinical low-resolution CT. Differences in trabecular architecture within acetabulum and femoral head were found between the fracture and control groups. In addition, the trabeculae within the femoral head of the cases with acetabular fracture differed in structure, density and texture with their corresponding control side femurs. These results suggest that lower density both at acetabulum and at femoral head, in combination with abnormal structure and texture at the femoral head, are associated with low-energy acetabular fractures in elderly subjects. We also demonstrated that machine learning approach can discriminate acetabular fracture subjects from controls using trabecular architecture and/or PFG as input variables. There is a gap in research that investigate the etiology of low-energy acetabular fractures. In this study, using clinically available data, we attempted to address some of the associated structural risk factors. Future work is still needed to further investigate the trabecular micro-architecture at higher resolutions, and the independent role of trabecular architecture beyond BMD.

## Electronic supplementary material

Below is the link to the electronic supplementary material.

Supplementary material 1 (PDF 1116 kb)

## References

[1] Bauer JS, Link TM, Burghardt A, Henning TD, Mueller D, Majumdar S, Prevrhal S (2007). Analysis of trabecular bone structure with multidetector spiral computed tomography in a simulated soft-tissue environment. Calcif. Tissue Int..

[2] Chiba K, Burghardt AJ, Osaki M, Majumdar S (2013). Heterogeneity of bone microstructure in the femoral head in patients with osteoporosis: an ex vivo HR-pQCT study. Bone.

[3] Crane GJ, Fazzalari NL, Parkinson IH, Vernon-Roberts B (1990). Age-related changes in femoral trabecular bone in arthrosis. Acta Orthop. Scand..

[4] de Joode S, Kalmet P, Fiddelers A, Poeze M, Blokhuis T (2019). Long-term functional outcome after a low-energy hip fracture in elderly patients. J. Orthop. Traumatol..

[5] Donohue D, Decker S, Ford J, Foley R, Dunbar K, Kumm T, Achors K, Mir H (2018). Opportunistic CT screening for osteoporosis in patients with pelvic and acetabular trauma: technique and potential clinical impact. J. Orthop. Trauma.

[6] Dougherty G (2001). A comparison of the texture of computed tomography and projection radiography images of vertebral trabecular bone using fractal signature and lacunarity. Med. Eng. Phys..

[7] Dougherty G, Henebry GM (2002). Lacunarity analysis of spatial pattern in CT images of vertebral trabecular bone for assessing osteoporosis. Med. Eng. Phys..

[8] Fazzalari NL, Parkinson IH (1998). Femoral trabecular bone of osteoarthritic and normal subjects in an age and sex matched group. Osteoarthr. Cartil..

[9] Ferguson TA, Patel R, Bhandari M, Matta JM (2010). Fractures of the acetabulum in patients aged 60 years and older: an epidemiological and radiological study. J. Bone Jt. Surg. Br..

[10] Firoozabadi R, Cross WW, Krieg JC, ChipRoutt MLJ (2017). Acetabular Fractures in the senior population—epidemiology, mortality and treatments. Arch. Bone Jt. Surg..

[11] Friedman J, Hastie T, Tibshirani R (2010). Regularization paths for generalized linear models. J. Stat. Softw..

[12] Gebre RK, Hirvasniemi J, Lantto I, Saarakkala S, Leppilahti J, Jämsä T (2019). Structural risk factors for low-energy acetabular fractures. Bone.

[13] Gelman A, Jakulin A, Pittau MG, Su YS (2008). A weakly informative default prior distribution for logistic and other regression models. Ann. Appl. Stat..

[14] Gnudi S, Ripamonti C, Lisi L, Fini M, Giardino R, Giavaresi G (2002). Proximal femur geometry to detect and distinguish femoral neck fractures from trochanteric fractures in postmenopausal women. Osteoporos. Int..

[15] Goldstein SA, Goulet R, McCubbrey D (1993). Measurement and significance of three-dimensional architecture to the mechanical integrity of trabecular bone. Calcif. Tissue Int..

[16] Guerado E, Cano JR, Cruz E (2012). Fractures of the acetabulum in elderly patients: an update. Injury.

[17] Haralick RM (1979). Statistical and structural approaches to texture. Proc. IEEE.

[18] Haralick RM, Shanmugam K, Dinstein I (2007). Textural features for image classification. IEEE Trans. Syst. Man. Cybern..

[19] Hartholt KA, Lee R, Burns ER, van Beeck EF (2019). Mortality from falls among US adults aged 75 years or older, 2000–2016. Jama.

[20] Hastie, T., R. Tibshirani, and J. Friedman. The Elements of Statistical Learning The Elements of Statistical Learning. 2017, pp. 241–256.

[21] Hildebrand T, Laib A, Mu R (1999). Direct three-dimensional morphometric analysis of. J. Bone Miner. Res..

[22] Hirvasniemi J, Niinimäki J, Thevenot J, Saarakkala S (2019). Bone density and texture from minimally post-processed knee radiographs in subjects with knee osteoarthritis. Ann. Biomed. Eng..

[23] Hirvasniemi J, Thevenot J, Kokkonen HT, Finnilä MA, Venäläinen MS, Jämsä T, Korhonen RK, Töyräs J, Saarakkala S (2016). Correlation of subchondral bone density and structure from plain radiographs with micro computed tomography ex vivo. Ann. Biomed. Eng..

[24] Janvier T, Jennane R, Valery A, Harrar K, Delplanque M, Lelong C, Loeuille D, Toumi H, Lespessailles E (2017). Subchondral tibial bone texture analysis predicts knee osteoarthritis progression: data from the Osteoarthritis Initiative: tibial bone texture & knee OA progression. Osteoarthr. Cartil..

[25] Kanis JA, Johnell O (2005). Requirements for DXA for the management of osteoporosis in Europe. Osteoporos. Int..

[26] Karhula, S. S., M. A. J. Finnilä, S. J. O. Rytky, D. M. Cooper, J. Thevenot, M. Valkealahti, K. P. H. Pritzker, M. Haapea, A. Joukainen, P. Lehenkari, H. Kröger, R. K. Korhonen, H. J. Nieminen, and S. Saarakkala. Quantifying subresolution 3D morphology of bone with clinical computed tomography. *Ann. Biomed. Eng.* 1–11, 2019. 10.1007/s10439-019-02374-2 PMC6949315 31583552

[27] Kim YS, Lee S, Sung YK, Lee BG (2016). Assessment of osteoporosis using pelvic diagnostic computed tomography. J. Bone Miner. Metab..

[28] Kuhn Max (2005). Building predictive models in R using the caret package. J. Stat. Softw..

[29] Lang TF, Guglielmi G, Van Kuijk C, De Serio A, Cammisa M, Genant HK (2002). Measurement of bone mineral density at the spine and proximal femur by volumetric quantitative computed tomography and dual-energy x-ray absorptiometry in elderly women with and without vertebral fractures. Bone.

[30] Letournel, E., Judet, R. Fractures of the acetabulum. Library of Congress, 1993, p. 736.

[31] Odgaard A (1997). Three-dimensional methods for quantification of cancellous bone architecture. Bone.

[32] Otsu N, Smith P, Reid DB, Environment C, Palo L, Alto P, Smith PL (1979). A threshold selection method from gray-level histograms. IEEE Trans. Syst. Man. Cybern..

[33] Pagenkopf E, Grose A, Partal G, Helfet DL (2006). Acetabular fractures in the elderly: treatment recommendations. HSS J..

[34] Pulkkinen P, Eckstein F, Lochmüller EM, Kuhn V, Jämsä T (2006). Association of geometric factors and failure load level with the distribution of cervical vs. trochanteric hip fractures. J. Bone Miner. Res..

[35] Pulkkinen P, Partanen J, Jalovaara P, Nieminen MT, Jämsä T (2011). Combination of radiograph-based trabecular and geometrical parameters can discriminate cervical hip fractures from controls in individuals with BMD in non-osteoporotic range. Bone.

[36] Robin X, Turck N, Hainard A, Tiberti N, Lisacek F, Mueller M, Sanchez J-C (2011). pROC: an open-source package for R and S+ to analyze and compare ROC curves. BMC Bioinform..

[37] Rothman KJ (1990). No adjustments are needed for multiple comparisons. Epidemiology.

[38] Shafiq-Ul-Hassan M, Zhang GG, Latifi K, Ullah G, Hunt DC, Balagurunathan Y, Abdalah MA, Schabath MB, Goldgof DG, Mackin D, Court LE, Gillies RJ, Moros EG (2017). Intrinsic dependencies of CT radiomic features on voxel size and number of gray levels. Med. Phys..

[39] Showalter C, Clymer BD, Richmond B, Powell K (2006). Three-dimensional texture analysis of cancellous bone cores evaluated at clinical CT resolutions. Osteoporos. Int..

[40] Sidey-gibbons JAM, Sidey-gibbons CJ (2019). Machine learning in medicine : a practical introduction.. BMC Med. Res. Methodol..

[41] Soh L, Tsatsoulis C, Member S (1999). Texture analysis of SAR sea ice imagery. IEEE Trans. Geosci. Remote Sens..

[42] Thevenot J, Hirvasniemi J, Finnilä M, Pulkkinen P, Kuhn V, Link T, Eckstein F, Jämsä T, Saarakkala S (2013). Trabecular homogeneity index derived from plain radiograph to evaluate bone quality. J. Bone Miner. Res..

[43] Thevenot J, Hirvasniemi J, Pulkkinen P, Määttä M, Korpelainen R, Saarakkala S, Jämsä T (2014). Assessment of risk of femoral neck fracture with radiographic texture parameters: a retrospective study. Radiology.

[44] Yang J, Mackin D, Jones AK, Court L, Zhang L, Ng CS, Fave X (2018). Harmonizing the pixel size in retrospective computed tomography radiomics studies. PLoS ONE.

[45] Zou H, Hastie T (2005). Regularization and variable selection via the elastic net. J. R. Stat. Soc. B.

