# Supplementary material for: Discrimination of Low-Energy Acetabular Fractures from Controls Using Computed Tomography-Based Bone Characteristics
Source: Ann Biomed Eng. 2020 Jul 9;49(1):367–81. doi: 10.1007/s10439-020-02563-4 (PMC7773622; doi:10.1007/s10439-020-02563-4)

Supplementary Table 1. Pearson bivariate correlation coefficients between bone volume fraction (V1), gray level co-occurrence matrix (GLCM) texture (V2 – V10) and gray value (GV) histogram (V11 - V15) variables measured from the acetabular region (AR).

Variables	V1	V2	V3	V4	V5	V6	V7	V8	V9	V10	V11	V12	V13	V14
**V15**	-0.563**	-0.541**	0.780**	-0.172*	0.855**	-0.702**	-0.361**	-0.559**	0.865**	-0.136	-0.597**	-0.367**	0.897**	-0.367**
**V14**	-0.232**	0.729**	-0.414**	0.332**	0.583**	0.641**	0.781**	0.863**	0.338**	-0.200**	0.237**	0.989**	-0.104	
**V13**	-0.826**	-0.392**	0.735**	-0.031	-0.749**	-0.563**	-0.145**	-0.350**	0.810**	-0.245**	-0.592**	-0.080		
**V12**	-0.263**	0.713**	-0.409**	0.343**	0.581**	0.638**	0.770**	0.839**	-0.339**	-0.211**	0.256**			
**V11**	0.356**	0.426**	-0.621**	0.237**	0.689**	0.548**	0.255**	0.504**	-0.653**	0.106				
**V10**	0.273**	0.337**	-0.557**	-0.834**	0.244**	0.437**	0.171*	-0.204**	-0.300**					
**V9**	-0.536**	-0.589**	0.885**	-0.078	-0.909**	-0.770**	-0.373**	-0.546**						
**V8**	-0.006	0.767**	-0.577**	0.455**	0.768**	0.734**	0.735**							
**V7**	-0.172*	0.940**	-0.564**	-0.113	0.620**	0.808**								
**V6**	0.216**	0.943**	-0.932**	-0.140	0.923**									
**V5**	0.399**	0.805**	-0.935**	0.125										
**V4**	-0.094	-0.153*	0.181*											
**V3**	-0.452**	-0.792**												
**V2**	0.071													

* Correlation is significant at the 0.05 level (2-tailed). ** Correlation is significant at the 0.01 level (2-tailed)

V1 = Bone volume fraction (BV/TV)
V2 = Contrast
V3 = Homogeneity
V4 = Correlation
V5 = Entropy
V6 = Difference entropy
V7 = Difference variance
V8 = Sum variance
V9 = Maximum probability
V10 = Information measure of correlation
V11 = GV mean
V12 = GV standard deviation
V13 = GV skewness
V14 = GV variance
V15 = GV kurtosis

Supplementary Table 2. Pearson bivariate correlation coefficients between bone volume fraction (V1), gray level co-occurrence matrix (GLCM) texture (V2 – V10) and gray value (GV) histogram (V11 - V15) variables measured from the femoral head region FHR-1.

Variables	V1	V2	V3	V4	V5	V6	V7	V8	V9	V10	V11	V12	V13	V14
**V15**	-0.203**	-0.337**	0.438**	-0.423**	-0.350**	0.238**	-0.670**	-0.663**	0.372**	0.287**	-0.344**	-0.524**	0.268**	-0.514**
**V14**	0.107	0.624**	-0.465**	0.278**	0.599**	0.599**	0.677**	0.865**	-0.022	-0.212**	0.316**	0.991**	-0.186**	
**V13**	-0.914**	-0.087	0.072	-0.184**	-0.083	-0.068	-0.083	-0.250**	-0.187**	0.135	-0.695**	-0.177*		
**V12**	0.095	0.621**	-0.481**	0.274**	0.604**	0.595**	0.697**	0.852**	-0.051	-0.209**	0.326**			
**V11**	0.549**	0.291**	-0.311**	0.185**	0.337**	0.272**	0.377**	0.456**	0.046	-0.041				
**V10**	-0.139*	0.456**	-0.573**	-0.881**	0.535**	0.427**	0.172*	-0.344**	-0.178*					
**V9**	0.167*	-0.094	0.493**	0.040	-0.147*	0.071	-0.531**	0.012						
**V8**	0.158*	0.562**	-0.441**	0.471**	0.568**	0.534**	0.721**							
**V7**	0.008	0.739**	-0.874**	0.056	0.808**	0.628**								
**V6**	0.001	0.970**	-0.688**	-0.418**	0.918**									
**V5**	0.012	0.955**	-0.891**	-0.384**										
**V4**	0.135	-0.410**	0.363**											
**V3**	-0.012	-0.819**												
**V2**	0.026													

* Correlation is significant at the 0.05 level (2-tailed). ** Correlation is significant at the 0.01 level (2-tailed)

V1 = Bone volume fraction (BV/TV)
V2 = Contrast
V3 = Homogeneity
V4 = Correlation
V5 = Entropy
V6 = Difference entropy
V7 = Difference variance
V8 = Sum variance
V9 = Maximum probability
V10 = Information measure of correlation
V11 = GV mean
V12 = GV standard deviation
V13 = GV skewness
V14 = GV variance
V15 = GV kurtosis

Supplementary Table 3. Pearson bivariate correlation coefficients between bone volume fraction (V1), gray level co-occurrence matrix (GLCM) texture (V2 – V10) and gray value (GV) histogram (V11 - V15) variables measured from the femoral head region FHR-2.

Variables	V1	V2	V3	V4	V5	V6	V7	V8	V9	V10	V11	V12	V13	V14
**V15**	0.147*	0.572**	-0.243**	-0.289**	-0.344**	-0.076	-0.768**	-0.497**	0.764**	0.142*	0.120	-0.411**	0.266**	-0.400**
**V14**	0.165*	-0.371**	0.525**	0.247**	0.454**	0.488**	0.539**	0.804**	-0.153*	-0.242**	0.128	0.988**	-0.197**	
**V13**	-0.908**	-0.194**	-0.045**	-0.208**	-0.005**	-0.150*	0.139*	-0.278**	-0.433**	0.178*	-0.676**	-0.197**		
**V12**	0.161*	-0.372**	0.506**	0.266**	0.450**	0.474**	0.552**	0.793**	-0.167*	-0.251**	0.134			
**V11**	0.569**	-0.140*	0.248**	0.092	0.312**	0.305**	0.161*	0.365**	0.195**	0.080				
**V10**	-0.214**	-0.583**	0.501**	-0.844**	0.619**	0.466**	0.217**	-0.291**	-0.145*					
**V9**	0.292**	0.664**	-0.190**	-0.029	-0.333**	-0.005**	-0.752**	-0.165**						
**V8**	0.244**	-0.430**	0.508**	0.428**	0.517**	0.466**	0.651**							
**V7**	-0.099	-0.892**	0.646**	0.032	0.774**	0.500**								
**V6**	0.075	-0.597**	0.957**	-0.488**	0.858**									
**V5**	-0.039	0.885**	0.917**	-0.439**										
**V4**	0.221**	0.358**	-0.489**											
**V3**	-0.006	-0.762**												
**V2**	0.174*													

* Correlation is significant at the 0.05 level (2-tailed). ** Correlation is significant at the 0.01 level (2-tailed)

V1 = Bone volume fraction (BV/TV)
V2 = Contrast
V3 = Homogeneity
V4 = Correlation
V5 = Entropy
V6 = Difference entropy
V7 = Difference variance
V8 = Sum variance
V9 = Maximum probability
V10 = Information measure of correlation
V11 = GV mean
V12 = GV standard deviation
V13 = GV skewness
V14 = GV variance
V15 = GV kurtosis

Supplementary Table 4. Correlation of trabecular architectural variables between the two femur head regions, FHR-1 and FHR-2, on femurs the side of the acetabular fractures and the corresponding control side femurs. Pearson correlation coefficients (r) and mean statistical test p-values for all subjects and individual genders are also shown.

Variables	Fracture side						Control side					
	All (n = 196)		F (n = 44)		M (n = 152)		All (n = 214)		F (n = 50)		M (n = 164)	
	r	*p*	*r*	*p*	*r*	*p*	*r*	*p*	*r*	*p*	*r*	*p*
**BV/TV**	0.404^{**}	0.123	0.528^{*}	0.221	0.367^{*}	0.239	0.577^{**}	0.001‡	0.441^{*}	0.103	0.566^{**}	0.002‡
**Contrast**	0.567^{**}	0.001‡†	0.671^{**}	0.011‡†	0.537^{**}	0.010‡†	0.735^{**}	< 0.001‡†	0.670^{**}	0.002‡†	0.755^{**}	< 0.001‡†
**Homogeneity**	0.563^{**}	< 0.001‡†	0.674^{**}	0.007‡†	0.523^{**}	< 0.001‡†	0.534^{**}	< 0.001‡†	0.562^{**}	<0.001‡	0.531^{**}	< 0.001‡†
**Correlation**	0.611^{**}	0.571	0.722^{**}	0.453	0.582^{**}	0.799	0.537^{**}	0.053	0.599^{**}	0.211	0.524^{**}	0.115
**Entropy**	0.599^{**}	0.004‡†	0.664^{**}	0.023‡†	0.579^{**}	0.034‡	0.643^{**}	< 0.001‡	0.661^{**}	0.003‡†	0.638^{**}	< 0.001‡
**Difference entropy**	0.569^{**}	0.051	0.650^{**}	0.044‡†	0.550^{**}	0.279	0.750^{**}	0.001‡†	0.733^{**}	0.032‡†	0.765^{**}	0.006‡†
**Difference variance**	0.462^{**}	< 0.001‡	0.598^{**}	0.024‡†	0.427^{**}	0.001‡	0.484^{**}	< 0.001‡†	0.595^{**}	0.009‡	0.460^{**}	< 0.001‡†
**Sum variance**	0.370^{**}	< 0.001‡†	0.545^{**}	0.012‡	0.343^{**}	0.006‡†	0.595^{**}	0.001‡†	0.539^{**}	0.070	0.600^{**}	0.007‡†
**Maximum probability**	0.258^{*}	< 0.001‡†	0.481^{*}	0.013‡†	0.201	< 0.001‡†	0.573^{**}	< 0.001‡†	0.651^{**}	0.017‡†	0.580^{**}	< 0.001‡†
**IMC**	0.670^{**}	0.907	0.754^{**}	0.725	0.645^{**}	0.996	0.557^{**}	0.0013†	0.640^{**}	0.022‡	0.541^{**}	0.008‡
**GV mean**	0.856^{**}	0.001‡	0.906^{**}	0.274	0.837^{**}	0.001‡	0.869^{**}	< 0.001‡	0.923^{**}	0.452	0.821^{**}	< 0.001‡†
**GV standard deviation**	0.620^{**}	< 0.001‡†	0.514^{*}	< 0.001‡	0.645^{**}	< 0.001‡†	0.679^{**}	< 0.001‡†	0.767^{**}	0.001‡	0.660^{**}	< 0.001‡†
**GV variance**	0.587^{**}	< 0.001‡†	0.466^{*}	< 0.001‡†	0.612^{**}	< 0.001‡†	0.686^{**}	< 0.001‡†	0.759^{**}	0.001‡	0.674^{**}	< 0.001‡†
**GV skewness**	0.484^{**}	0.294	0.474^{*}	0.675	0.470^{**}	0.330	0.642^{**}	0.009‡†	0.646^{**}	0.400	0.606^{**}	0.016‡†
**GV kurtosis**	0.259^{**}	0.000‡†	0.253	0.002‡†	0.262^{*}	< 0.001‡†	0.236^{**}	< 0.001‡†	0.499^{*}	0.041‡†	0.220^{*}	< 0.001‡†

*Correlation is significant at the 0.05 level (2-tailed), **Correlation is significant at the 0.01 level (2-tailed)
‡Parametric Independent samples t-test ($p < 0.05$). ‡†Nonparametric Mann-Whitney U-test ($p < 0.05$),
BV/TV = Bone volume fraction, IMC = Information measure of correlation, GV = Gray values, SD = Standard deviation

Supplementary Table 5. Proximal femur geometry variables neck shaft angle (NSA) and femoral neck axis length (FNALa and FNALb). Values given as means, standard deviations (SD) and 95% confidence interval [CI]. Statistical p-values of the differences for all subjects and individual genders are also shown.

Variables	Fracture side			Control side			*p*		
	All (n = 95)	F (n = 20)	M (n = 75)	All (n = 103)	F (n=25)	M (n = 78)	All (n=198)	F (n=45)	M (n = 153)
**NSA (°)**	121.71 (5.56) [120.58-122.84]	118.76 (6.49) [115.72-121.79]	122.49 (5.05) [121.33-123.66]	124.60 (5.64) [123.49-125.70]	124.51 (5.85) [122.09-126.93]	124.63 (5.61) [123.36-125.89]	<0.001‡	0.003†	0.015†
**FNALb (mm)**	78.36 (0.36) [77.14-79.58]	71.74 (4.05) [70.04-73.83]	80.07 (5.24) [78.87-81.28]	76.03 (0.03) [74.75-77.30]	70.59 (5.04) [68.51-73.83]	77.77 (5.98) [76.42-79.12]	0.010‡	0.615	0.037‡†
**FNALa (mm)**	103.46 (6.92) [102.06-104.87]	94.98 (4.34) [92.95-97.01]	105.73 (5.59) [104.44-107.01]	101.22 (8.25) [99.61-102.83]	93.37 (6.20) [90.81-95.93]	103.74 (7.19) [102.12-105.36]	0.073	0.749	0.125

‡Parametric Independent samples t-test ($p < 0.05$). ‡†Nonparametric Mann-Whitney U-test ($p < 0.05$)

Supplementary Table 6. Coefficient weights of the variables used in the final EN model of trabecular architecture features at the acetabular region (AR). The trabecular architecture features were bone volume fracture (BV/TV), gray level co-occurrence matrix and gray value (GV) histogram variables.

Model inputs	All subjects	Females	Males
**Intercept**	0.000	0.002	-0.002
**Correlation**	-	-	0.257
**Difference variance**	-	0.343	-
**GV mean**	0.119	0.111	0.353

Supplementary Table 7. Elastic net (EN) regression hyperparameters (α, λ) used in the acetabular region (AR) and femoral head region (FHR) final models for all subjects, females and males. Models with and without proximal femur geometry (PFG) are shown.

Model	Without PFG (α, λ)	With PFG (α, λ)
AR	0.97, 0.116	-
AR-female	0.94, 0.091	-
AR-male	0.97, 0.056	-
FHR-1	0.01, 0.126	0.01, 0.026
FHR-1-female	0.94, 0.006	0.82, 0.146
FHR-1-male	0.1, 0.146	0.01, 0.031
FHR-2	0.01, 0.141	0.97, 0.011
FHR-2-female	0.73, 0.056	0.01, 0.016
FHR-2-male	1.00, 0.036	0.97, 0.026
FHR-1&2	0.88, 0.026	0.91, 0.021
FHR-1&2 - female	0.94, 0.136	0.10, 0.001
FHR-1&2 - male	0.91, 0.031	0.13, 0.146

Supplementary Figure 1. Receiver operating characteristics curves of the femur head region 1 (FHR-1) and femur head region 2 (FHR-2). Bayesian logistic regression (BLR) and elastic net (EN) models for all subjects and individual genders. FHR was measured from the fracture side. (A) and (B) show FHR-1 curves without and with PFG, while (C) and (D) show FHR-2 curves without and with PFG.

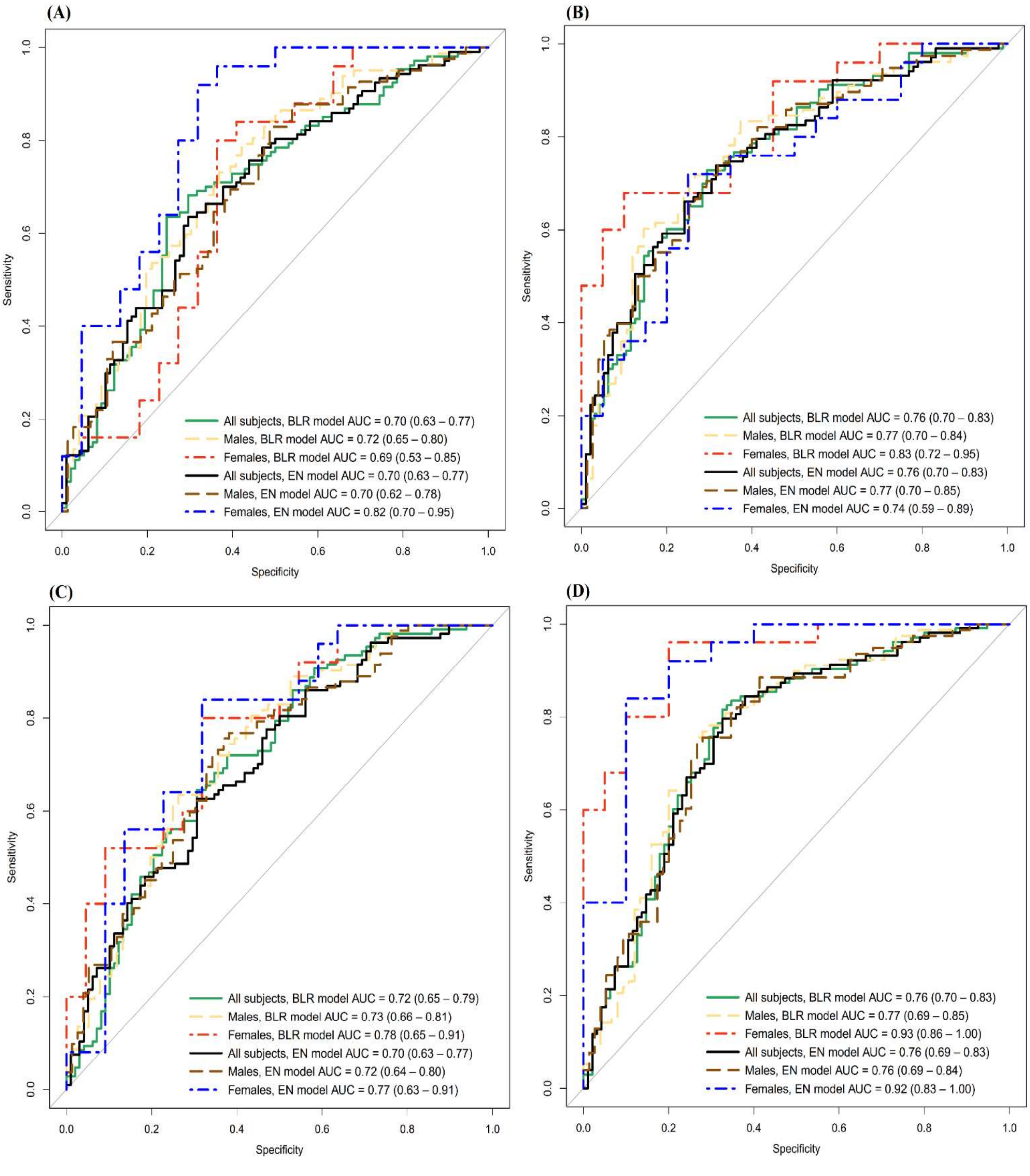

Supplement: Supplementary file 1 — Supplementary material 1 (PDF 1116 kb) [file 10439_2020_2563_MOESM1_ESM.pdf]
